# Caloric restriction mitigates age-associated senescence characteristics in subcutaneous adipose tissue-derived stem cells

**DOI:** 10.18632/aging.205812

**Published:** 2024-05-09

**Authors:** Somaiah Chinnapaka, Hamid Malekzadeh, Zayaan Tirmizi, Asim Ejaz

**Affiliations:** 1 Department of Plastic Surgery, University of Pittsburgh, Pittsburgh, PA 15261, USA

**Keywords:** adipose stem cells, adipose tissue, stemness, differentiation, autophagy, senescence, caloric restriction, reactive oxygen species

## Abstract

Adipose tissue regulates metabolic balance, but aging disrupts it, shifting fat from insulin-sensitive subcutaneous to insulin-resistant visceral depots, impacting overall metabolic health. Adipose-derived stem cells (ASCs) are crucial for tissue regeneration, but aging diminishes their stemness and regeneration potential. Our findings reveal that aging is associated with a decrease in subcutaneous adipose tissue mass and an increase in the visceral fat depots mass. Aging is associated with increase in adipose tissue fibrosis but no significant change in adipocyte size was observed with age. Long term caloric restriction failed to prevent fibrotic changes but resulted in significant decrease in adipocytes size. Aged subcutaneous ASCs displayed an increased production of ROS. Using mitochondrial membrane activity as an indicator of stem cell quiescence and senescence, we observed a significant decrease in quiescence ASCs with age exclusively in subcutaneous adipose depot. In addition, aged subcutaneous adipose tissue accumulated more senescent ASCs having defective autophagy activity. However, long-term caloric restriction leads to a reduction in mitochondrial activity in ASCs. Furthermore, caloric restriction prevents the accumulation of senescent cells and helps retain autophagy activity in aging ASCs. These results suggest that caloric restriction and caloric restriction mimetics hold promise as a potential strategy to rejuvenate the stemness of aged ASCs. Further investigations, including *in* *vivo* evaluations using controlled interventions in animals and human studies, will be necessary to validate these findings and establish the clinical potential of this well-established approach for enhancing the stemness of aged stem cells.

## INTRODUCTION

Adipose tissue plays a crucial and multifaceted role in maintaining overall metabolic balance, insulin sensitivity, and immune modulation. Its primary function involves serving as a reservoir for excess fatty acids, preventing their accumulation in other organs, and avoiding the associated negative consequences, such as steatosis and lipotoxicity [1]. However, as individuals age, there is a progressive deterioration of cellular function, making aging one of the greatest risk factors for the development of numerous debilitating diseases [2]. Among these age-related conditions, insulin resistance stands out as a prevalent metabolic disorder affecting a significant portion of the elderly population in the United States. This condition is associated with increased mortality rates, reduced functional status, and higher hospitalization risk, and places a substantial burden on healthcare systems [3]. Although the connection between obesity and diabetes is well-established, the relationship between aging and type 2 diabetes remains less understood [4]. In fact, the correlation between declining glucose tolerance and aging was first observed nearly a century ago in 1921 [5]. Since then, numerous studies have confirmed that reduced insulin sensitivity is the primary contributor to age-related impairments in glucose metabolism [6, 7].

To better understand the impact of aging on adipose tissue, it is essential to recognize that adipose tissue can be categorized into two main depots based on its anatomical localization: subcutaneous and visceral depots. These depots differ in their insulin sensitivity, with subcutaneous adipose tissue displaying higher insulin sensitivity compared to the more insulin-resistant visceral depot [8]. However, with advancing age, there is a gradual loss of subcutaneous adipose tissue volume, leading to diminished glucose and lipid uptake. This phenomenon is known as “lipid overflow hypothesis,” which results in the ectopic deposition of lipids in muscles and the liver, ultimately contributing to the development of insulin resistance [9, 10] Understanding the complex interplay between aging, adipose tissue distribution, and insulin sensitivity is crucial for developing effective strategies to combat age-associated metabolic disorders.

Stromal Vascular Fraction (SVF) is a complex mixture of various cell types found in adipose tissue, and it holds particular significance due to the presence of adipose-derived stem cells (ASCs) among its constituents [11, 12]. These ASCs exhibit a distinct immune-phenotypic profile, characterized by the expression of specific cell surface markers such as CD34^+^, CD90^+^, CD29^+^, CD45^−^, and CD31^−^, which identify them as mesenchymal cells with the potential to differentiate into adipocytes, bone cells, and cartilage cells [13, 14]. The microenvironment of white adipose tissue houses these ASCs predominantly within the vascular stroma surrounding small blood vessels, facilitating their proliferation and adipogenic differentiation [13]. At the transcriptional level, the primary regulators of adipogenesis are the key transcription factors peroxisome proliferator-activated receptor γ (PPARγ), CCAAT/enhancer binding protein α (C/EBPα), and CCAAT/enhancer binding protein β [15]. These factors play essential roles in coordinating the expression of genes involved in lipid metabolism, leading to growth arrest and the production of adipocyte-specific hormones like adiponectin and leptin [16]. The regenerative properties of ASCs are vital for maintaining adipose tissue homeostasis and function. However, as individuals age, there is a decline in the stemness features of ASCs characterized by a decrease in their ability of maintaining quiescence, self-renewal and differentiation, which contributes to the age-related reduction in subcutaneous fat depot size [17–20]. Oxidative stress within adipose tissue during the aging process has been proposed as a potential factor negatively affecting the differentiation capacity of ASCs [21–23]. Yet, the precise mechanisms responsible for the loss of stemness in aging ASCs remain to be fully elucidated. Recent studies have shed light on a possible link between decreased autophagy (the process of cellular self-cleaning), increased activity of the mechanistic target of rapamycin (mTOR) pathway, and the accumulation of reactive oxygen species (ROS) with the loss of stemness and the induction of premature senescence in ASCs [24–26]. Understanding the mechanisms underlying the age-related decline in ASC stemness is crucial for developing strategies to mitigate the impact of age-related decline in ASCs stemness. Furthermore, investigating the potential therapeutic interventions that can preserve or restore the stemness properties of ASCs may hold promise for combating age-related adipose tissue dysfunction and its associated metabolic disorders.

Caloric restriction (CR) is a powerful and therapeutic nutritional intervention that involves a sustained reduction in calorie intake while ensuring adequate nutrition [27]. Extensive research has demonstrated its remarkable ability to extend lifespan and reduce the risk of age-related diseases across various species, from yeast to primates [25]. In humans, CR has shown numerous health benefits, including the prevention of cardiovascular disease, hypertension, obesity, type 2 diabetes, chronic inflammation, and certain cancers. This makes CR an intriguing and promising approach for promoting overall health and longevity [28]. One of the key contributors to the health-promoting effects of CR is its profound impact on metabolic function. CR induces a decrease in white adipose tissue (WAT) mass, leading to increased fatty acid oxidation and preventing the harmful ectopic deposition of lipids. Furthermore, CR enhances insulin sensitivity and improves glucose tolerance, factors crucial for maintaining metabolic health and reducing the risk of metabolic disorders [27].

Long-term CR has been found to result in reduced adipocyte size and a beneficial remodeling of body fat composition, shifting away from visceral white adipose tissue towards subcutaneous white adipose tissue. This shift is significant as subcutaneous fat tends to have positive effects on aging and obesity, whereas visceral is associated with detrimental health outcomes. Consequently, this transition contributes to the extension of healthspan, enhancing the overall quality of life as individuals age. Moreover, previous studies have revealed that bariatric surgery, a form of caloric restriction, can promote the stemness features of adipose-derived stem cells (ASCs) [25, 26]. This effect is achieved by inducing autophagy and reducing differentiation. Such findings highlight the regenerative potential of caloric restriction and suggest that it may play a crucial role in preserving the function and regenerative capacity of ASCs, which could have significant implications for regenerative medicine and age-related tissue repair.

In this study, we aimed to comprehensively characterize the alterations taking place in both subcutaneous and visceral adipose tissue, as well as in the resident adipose-derived stem cells (ASCs), with age. Additionally, we sought to investigate the potential impact of long-term caloric restriction in mitigating these age-related changes. Understanding these changes is essential, as they play a significant role in age-related metabolic dysregulation and the development of various health conditions.

## MATERIALS AND METHODS

### Mice

Female and male C57BL/6 mice and atg7 flox/flox mice [29] were used in this study. Animals were housed in an Association for Assessment and Accreditation of Laboratory Animal Care International (AAALAC)-approved facility and were treated according to the National Institutes of Health Guide for the Care and Use of Laboratory Animals. Ethical approval for all experiments was obtained from the University of Pittsburgh Institutional Animal Care and Use Committee (IACUC). Caloric-restricted group and ad-libitum group animals were fed as published [30]. Animals allocated to the caloric restricted cohort were introduced to the reduced caloric intake in a stepwise fashion over a period of 3 weeks, beginning at 14 weeks of age. Caloric restricted animals were fed 60% less calories compared to the ad libitum cohort [30].

### Isolation and cultivation of ASCs

The isolation of adipose precursor cells was performed as follows [31]. Adipose tissue biopsies obtained after surgical procedures were aseptically transferred to the laboratory in sterile sealed tubes. The tissue was rinsed with Dulbecco’s phosphate-buffered saline (PBS; Sigma-Aldrich, St. Louis, MO, USA). Subsequently, the tissue was cut into small pieces and subjected to digestion in a buffer solution (Hank’s Balanced Salt Solution (HBSS) containing 200 U/mL collagenase (CLS Type II, Worthington Biochemical Corp., Lakewood, NJ, USA) and 2% w/v BSA (Sigma-Aldrich, St. Louis, MO, USA)) under continuous stirring for 60 minutes at 37°C, using a ratio of 1 mg adipose tissue to 3 mL digestion buffer. Following digestion, the dispersed tissue was centrifuged at room temperature for 10 minutes at 200 g. The floating adipocytes were aspirated, and the remaining pelleted cells representing the stromal vascular fraction (SVF) were suspended in an erythrocyte lysis buffer (0.155 M NH4Cl, 5.7 mM K2HPO4, 0.1 mM EDTA, pH 7.3) and incubated for 10 minutes at room temperature. To eliminate tissue debris, the cell suspension was passed through a nylon mesh filter (100 μm pore size; Thermo Fisher Scientific, Waltham, MA, USA). The pelleted SVF obtained after another centrifugation step (10 minutes at 200 g) was resuspended in ASC medium (a mixture of Dulbecco’s Modified Eagle’s Medium (DMEM) and F-12 medium (1:1) with HEPES and L-glutamine; Thermo Fisher Scientific, Waltham, MA, USA), supplemented with 33 μM biotin, 17 μM pantothenate (both from Sigma-Aldrich, St. Louis, MO, USA), 10 ng/mL epidermal growth factor (EGF), 1 ng/mL basic fibroblast growth factor (bFGF), 500 ng/mL insulin, 2.5% fetal bovine serum, and 12.5 μM/mL gentamicin (all from Sigma-Aldrich, St. Louis, MO, USA). The SVF cells were seeded in T175 flasks at a density of 50,000 cells/cm2.

Once the cells reached 70% confluence, they were washed with PBS and detached using 0.05% trypsin-EDTA 1x solution (Sigma-Aldrich, St. Louis, MO, USA). The trypsin was inactivated by adding ASC medium supplemented with 10% FBS, and the cells were pelleted by centrifugation at 300 g for 5 minutes. Subsequently, the cells were seeded at a density of 5000 cells/cm2 and allowed to reach 70% confluence before further passaging. For this study, adipose-derived stem cells (ASCs) at passages 3–4 were used.

### Adipogenic differentiation

To initiate adipogenesis, ASCs were seeded at a density of 50,000 cells/cm^2^ in 6-well cell culture plates and allowed to reach confluence. The induction of adipogenesis was carried out using a differentiation medium consisting of 0.2 μM insulin, 0.5 mM 1-methyl-3-isobutylxanthine (IBMX), 0.25 μM dexamethasone, and 10 μg/mL transferrin, all obtained from Sigma-Aldrich, St. Louis, MO, USA. This differentiation medium was prepared by supplementing ASC medium. After 3 days of differentiation, the medium was replaced, and the cells were cultured in differentiation medium without IBMX for 14 days.

### ATG7 knock-down

ASCs isolated from atg7 flox/flox mice were transduced with cre-expressing lentivirus particles and selected using puromycin. Empty virus particles were used as controls. Knockdown efficiency was confirmed by Western blot analysis for atg7 expression.

### Oil red O staining

To visualize lipid droplets, the cells were fixed with a solution of 4% paraformaldehyde in PBS for 1 hour. Subsequently, they were stained with 0.3% Oil Red O (Sigma-Aldrich, St. Louis, MO, USA) dissolved in a mixture of isopropanol and water (60:40) for 1 hour. After staining, the cells were washed twice with distilled water to remove any excess stain.

### Reactive oxygen species detection

ROS levels were assessed using the fluorescent dye 2′,7′-dichlorofluorescin diacetate (DCFH2-DA) probe obtained from Invitrogen, Waltham, MA, USA. SVF cells were stained with 50 μM DCFH2-DA dye for 30 minutes at 37°C in the presence of anti-CD45, CD31, CD34, and DAPI. The stained cells were then analyzed using flow cytometry to examine and quantify the fluorescent signal indicative of ROS levels.

### TMRM staining

SVF cells were incubated at 37°C with anti-CD45, CD31, CD34, DAPI, and 100 nM TMRM (Sigma-Aldrich, St. Louis, MO, USA) for 30 min at 37°C. Stained cells were analyzed by flow cytometry.

### Senescence staining

Senescence-associated β-galactosidase activity was analyzed in SVF cells *ex vivo* using Imagene^™^ Green Kit (Thermo Fisher Scientific, Waltham, MA, USA) following the kit protocol.

### Autophagy analyses

Autophagy activity was analyzed in the SVF cells *ex vivo* using FlowCellect^™^ GFP-LC3 Reporter Autophagy Assay Kit (Millipore Sigma, USA) following the kit protocol.

### Western blot

Western blot analysis was conducted following a similar procedure as described previously [25]. The protein levels were normalized using the “Pierce BCA Protein Assay Kit” from Thermo Fisher Scientific, Waltham, MA, USA. Cell lysates containing 15 μg of total protein per lane were prepared using sodium dodecyl sulfate (SDS) sample buffer. Subsequently, the samples were separated by SDS-polyacrylamide gel electrophoresis (PAGE) and transferred onto polyvinylidene difluoride membranes. The membranes were then probed with the following antibodies: α-Tubulin (Proteintech, Rosemont, IL, USA), perilipin (Cell Signaling Technology, Danvers, MA, USA), ATG7 (Proteintech, USA), and rabbit anti-rat IgG HRP (Proteintech, USA). Densitometric analyses of the blots were performed using ImageJ software.

### Statistical analysis

Statistical analyses were performed in GraphPad Prism (GraphPad Software Inc., La Jolla, CA, USA). FlowJo was used for FACS analysis. The significance of the difference between means was assessed by the Student’s *t*-test or analysis of variance. Error bars are represented as the mean ± SEM. Values were significant at *p*-values of <0.05 (^*^p < 0.01 and ^#^*p* < 0.05).

## RESULTS

### Aging is associated with the redistribution of subcutaneous and visceral adipose tissue depots

**Figure 1:**
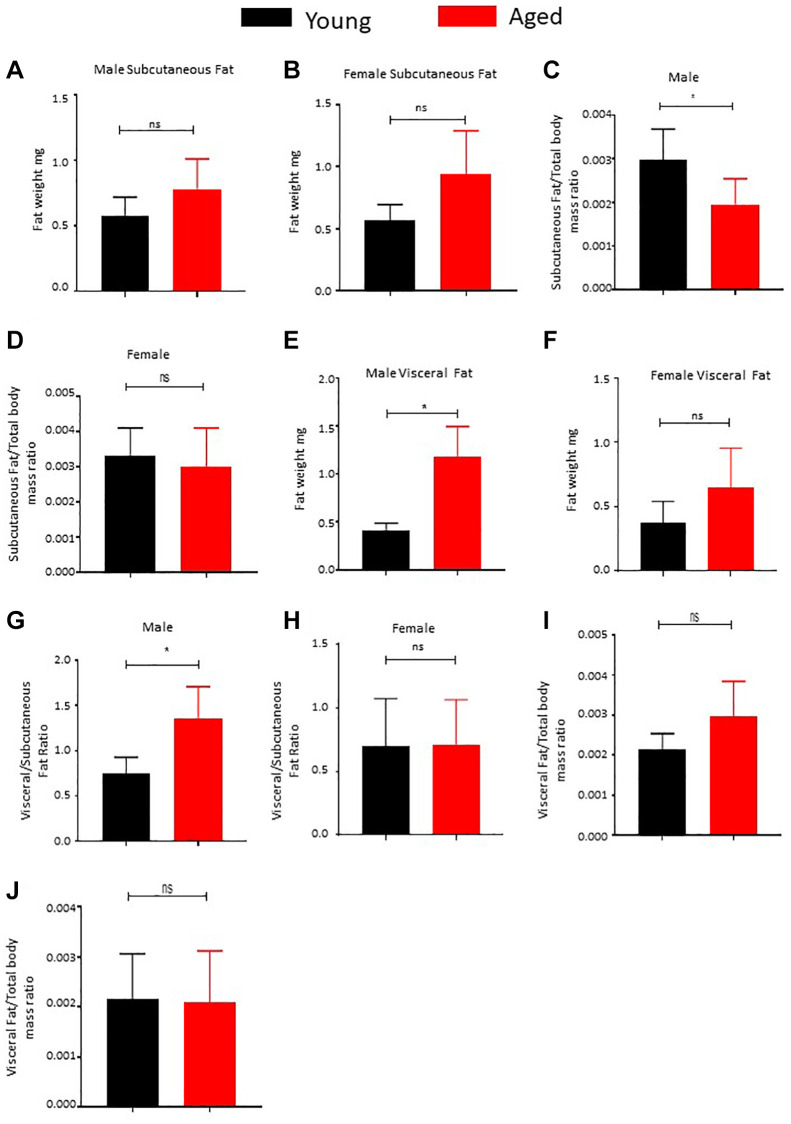
**Effect of age on body fat distribution.** (**A**, **B**) Subcutaneous adipose tissue was surgically excised from male (**A**) and female (**B**) mice and weighed (*n* = 5). (**C**, **D**) Subcutaneous fat and total body mass were measured and the ratio was calculated and plotted (*n* = 5). (**E**, **F**) Visceral fat was surgically excised from male (**E**) and female (**F**) mice and weighed (*n* = 5). (**G**, **H**) Ratio of subcutaneous to visceral fat mass was calculated for male (**G**) and female (**H**) mice and plotted. (**I**, **J**) Ratio of visceral fat mass to total body mass was calculated and plotted. *P* value < 0.05 = ^*^, ns = non-significant.

We conducted a comprehensive comparative analysis of subcutaneous and visceral adipose tissue fat mass in young (3–4 months) and aged (22–24 months) mice to shed light on distinct patterns observed in these adipose depots in relation to age and sex. Regarding subcutaneous adipose tissue, we found no significant alterations in fat mass between the young and aged male mice (Figure 1A) as well as the female mice (Figure 1B) cohorts, despite an increase in total body mass (Supplementary Figure 1A, 1B). Intriguingly, we observed a noteworthy decrease in the subcutaneous fat to total body mass ratio in male mice (Figure 1C), implying a significant limitation in the expansion of male subcutaneous adipose tissue proportionally to overall body mass. Conversely, the ratio change was not significant in the female mice cohort (Figure 1D). These findings suggest a restricted capacity of male subcutaneous adipose tissue to increase in size relative to the overall body.

In contrast, our examination of visceral fat depots yielded intriguing results. We observed a substantial increase in fat mass in male mice (Figure 1E), while female mice displayed a strong trend toward gaining visceral fat mass with age (Figure 1F). Additionally, we noted a significant rise in the ratio of male visceral to subcutaneous fat (Figure 1G) and a notable increase in visceral fat mass relative to the overall body mass increase in the male cohort (Figure 1I). Conversely, the female cohort did not exhibit a notable increase in the ratio of visceral to subcutaneous mass (Figure 1H), and the increase in the visceral depot among females was proportional to the overall body mass increase (Figure 1J). In summary, our analyzed data reveals a clear tendency of increased visceral fat mass with age, with this trend being significantly more prominent in male mice, who also demonstrated an inability to expand subcutaneous fat mass as they age.

### Aging result in fibrotic changes in adipose tissue

**Figure 2:**
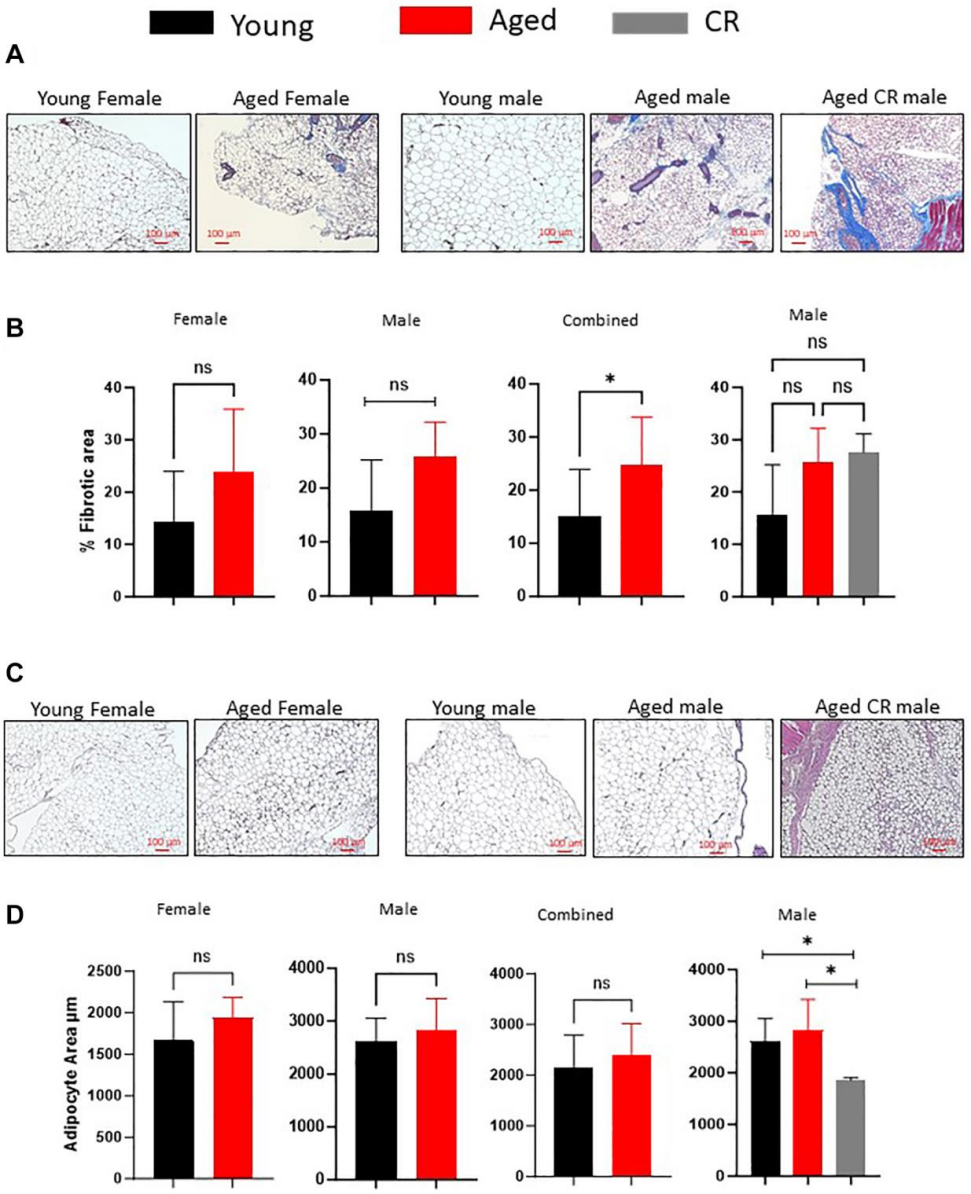
**Effect of aging on adipose tissue fibrosis and adipocyte size distribution.** (**A**, **B**) Masson’s Trichrome staining of adipose tissue sections (**A**) were quantified for staining intensity using ImageJ and plotted (**B**). (**C**, **D**) Adipose tissue sections from different mice groups were stained with H&E stain (**C**) and adipocytes size was measured using ImageJ software (**D**). (*n* = 3–5 mice per group) *P*-value < 0.05 = ^*^, ns = non-significant.

Aging correlates with increased adipose tissue fibrosis, as evidenced by Masson’s Trichrome staining for collagen fibers (Figure 2A, 2B). We investigated the impact of long-term caloric restriction (CR), a dietary regimen known to delay aging and promote healthspan, on adipose tissue fibrosis. Our findings revealed that age- and sex-matched mice subjected to long-term CR exhibited no significant alterations in fibrosis scores compared to controls (Figure 2A, 2B). Furthermore, we explored the influence of aging on adipocyte size. Interestingly, our analysis demonstrated no significant differences in adipocyte size between young and aged male and female mice (Figure 2C, 2D). Conversely, long-term caloric restriction led to a considerable reduction in adipocyte size among age- and sex-matched mice (Figure 2C, 2D), suggesting a regulatory effect of CR on adipocyte morphology.

### Aging is associated with oxidative stress in subcutaneous adipose tissue-resident ASCs

**Figure 3:**
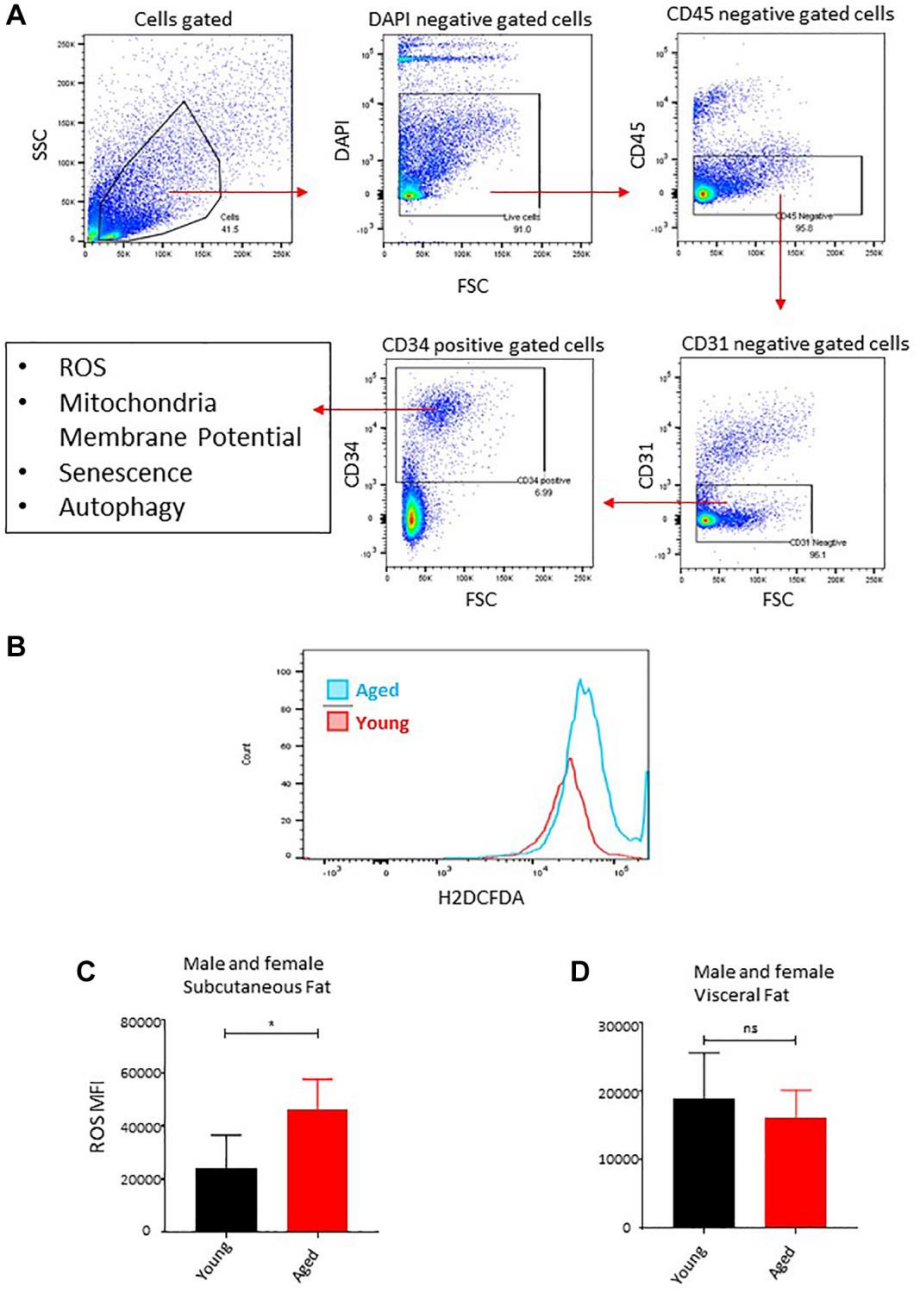
**Age-associated oxidative stress in ASCs.** (**A**) Flow cytometry gating strategy used in this study to gate on CD34^+^ fraction after excluding DAPI positive (dead cells), CD45^+^ (hematopoietic lineage), and CD31^+^ (endothelial) cells for downstream analysis of ROS, mitochondrial membrane potential, senescence, and autophagy markers. (**B**) ROS concentration was measured in ASCs. A representative histogram of flow cytometric measurement of DCFDA fluorescence in young and aged subcutaneous ASCs. (**C**, **D**) Subcutaneous ASCs (**C**) and visceral (**D**) were isolated, treated with DCFDA dye *ex vivo*, and analyzed by FACS. Graphs are representative of eight mice per group and ASCs from two mice were pooled together to achieve 5000 events count. (*n* = 2 male and 2 female mice per group) *P* value < 0.05 = ^*^, ns = non-significant.

Chronic inflammation, marked by ongoing secretion of proinflammatory factors above normal levels, plays a significant role in the process of aging and age-related diseases. Adipose tissue, particularly adipose-derived stem cells (ASCs), have been implicated as a significant contributor to inflammation during the aging process [21, 32]. Hyperactivity of mitochondria is linked to detrimental ROS levels, inflammation, and reduced adipogenesis [21, 33]. In this study, we analyzed the ROS levels in freshly isolated ASCs *ex vivo* among our young and aged mice cohorts. Flow cytometry was employed to analyze the ROS staining pattern in CD34^+^ cells by excluding the DAPI^+^ (dead cells), CD45^+^ (hematopoietic lineage cells), and CD31^+^ (endothelial cells) (Figure 3A). We used a well-established and recognized ROS measuring probe DCFDA and measured the fluorescence intensity by employing flow cytometry (Figure 3B). In line with our observation reflecting the failure of subcutaneous adipose tissue to expend with age, our ROS analyses showed increased ROS production by subcutaneous ASCs in aged mice (Figure 3C), while we observed no significant changes in ROS levels in the visceral ASCs (Figure 3D).

### Long-term caloric restriction prevents age-associated loss of ASCs quiescence in subcutaneous adipose tissue

**Figure 4:**
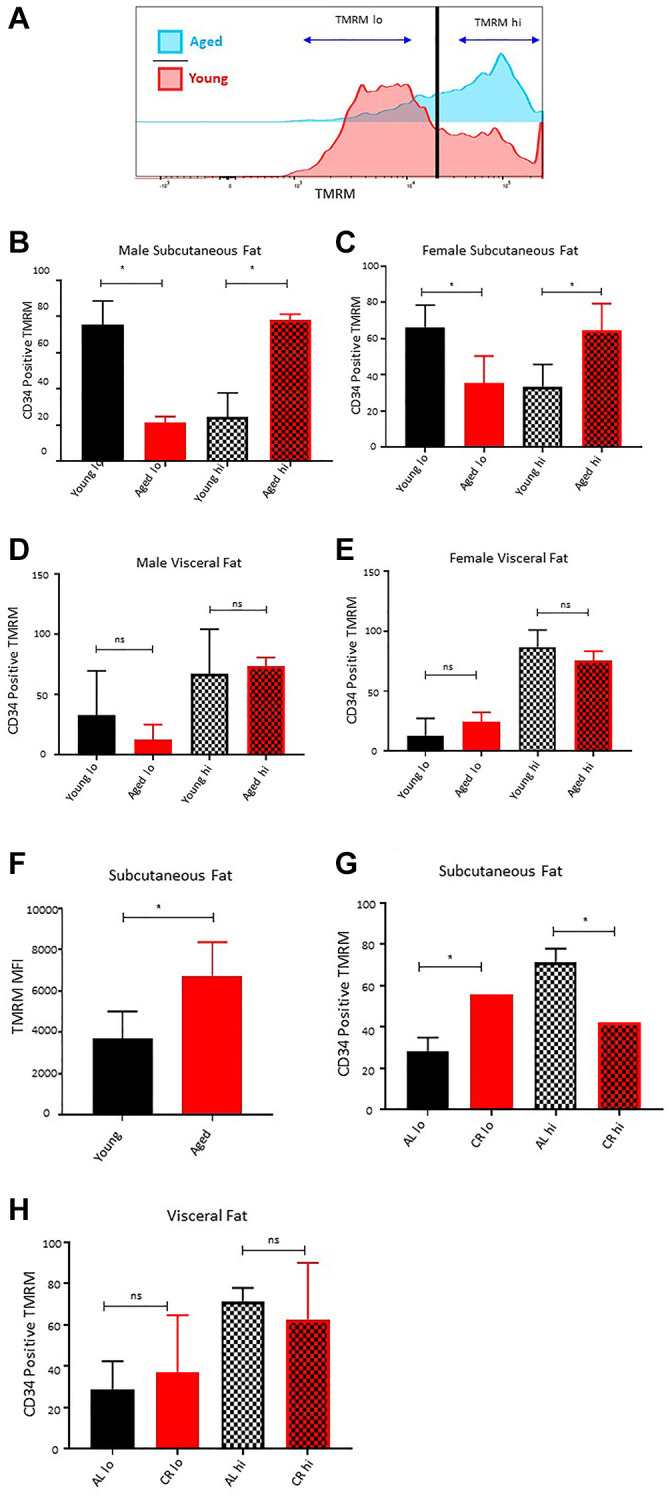
**Effect of age on ASCs quiescence.** (**A**) Representative histogram of TMRM staining in subcutaneous ASCs from young and aged mice. (**B**, **C**) Percentage of TMRM lo and TMRM hi ASCs in subcutaneous adipose tissue of young and aged male (**B**) and female (**C**) mice. (**D**, **E**) Percentage of TMRM lo and TMRM hi ASCs in visceral adipose tissue of young and aged male (**D**) and female (**E**) mice. (**F**) Mean fluorescence intensity of TMRM in young and aged ASCs. (**G**, **H**) Percentage of TMRM lo and TMRM hi ASCs in subcutaneous (**G**) and visceral (**H**) adipose tissue of mice fed on caloric restricted (CR) and ad-libitum (AL) diet. (*n* = 3–5 mice per group) *P*-value < 0.05 = ^*^, ns = non-significant.

Increased mitochondrial activity and ROS productions lead to the loss of quiescence, stemness, and increased differentiation of ASCs [21, 33, 34]. We compared the mitochondrial activity by measuring the mitochondrial membrane potential (TMRM staining) of freshly isolated CD34^+^ ASCs *ex vivo* in the subcutaneous and visceral adipose depots of young and aged mice. Flow cytometric analysis of TMRM staining revealed two distinct populations of ASCs based on TMRM fluorescence intensity, referred to as TMRM lo and TMRM hi (Figure 4A). Previous studies have shown that TMRM lo ASCs, cardiac progenitors, and hematopoietic stem cells represent quiescence stem cells having higher self-renewal potential and lower differentiation tendency [34, 35]. These TMRM lo cells play an important role in tissue regeneration and stress mitigation [34, 35]. Our flow cytometric data analyses revealed a significant decrease in the percentage of CD34^+^ TMRM lo cells with age in the subcutaneous adipose tissue in both male and female cohorts (Figure 4A–4C), while a significantly higher accumulation of TMRM hi ASCs was observed in both sexes with age (Figure 4B, 4C). These results demonstrate that aging is associated with the loss of quiescent ASCs in subcutaneous adipose tissue.

Furthermore, we analyzed the distribution of TMRM lo and TMRM hi cells in the ASCs isolated from visceral adipose tissue of young and aged mice. Interestingly, unlike the subcutaneous adipose tissue, there was no significant change in the distribution pattern of TMRM lo and TMRM hi ASCs in the visceral adipose compartment of aged mice compared to the young mice for both male and female mice cohorts (Figure 4D, 4E). Additionally, the average mean fluorescence intensity of TMRM stain in CD34^+^ ASCs was significantly higher in subcutaneous adipose tissue of aged mice (Figure 4F).

Previous studies have shown that long-term caloric restriction extends the life span in many species by protecting cells from oxidative damage, inducing DNA repair, and promoting autophagy [25]. Caloric restriction also preserves the age-associated decline in self-renewal and stemness in various stem cell populations, including hematopoietic stem cells, skin stem cells, intestinal stem cells, and muscle stem cells [36–39]. In this study, we utilized the metabolic readout of mitochondrial activity as a marker of stemness and quiescence to investigate the effect of caloric restriction on ASCs health. ASCs isolated from the subcutaneous adipose tissue of the 22 months old male mice maintained on a calorie-restricted diet showed a significantly higher percentage of CD34^+^ TMRM lo ASCs compared to the age and sex-matched mice kept on ad-libitum (Figure 4G). The percentage of TMRM lo in caloric-restricted mice was approximately similar to as observed in young mice. Conversely, there was a lower proportion of CD34^+^ TMRM hi ASCs in long-term caloric-restricted mice. We observed no significant differences in the distribution of CD34^+^ TMRM lo and hi cells in the visceral fat of young and caloric-restricted aged mice (Figure 4H). These results suggest that caloric restriction has the potential to preserve the age-dependent loss of CD34^+^ TMRM lo ASCs in subcutaneous adipose tissue.

### Long-term caloric restriction prevents accumulation of senescence ASCs in subcutaneous adipose tissue in aged mice

**Figure 5:**
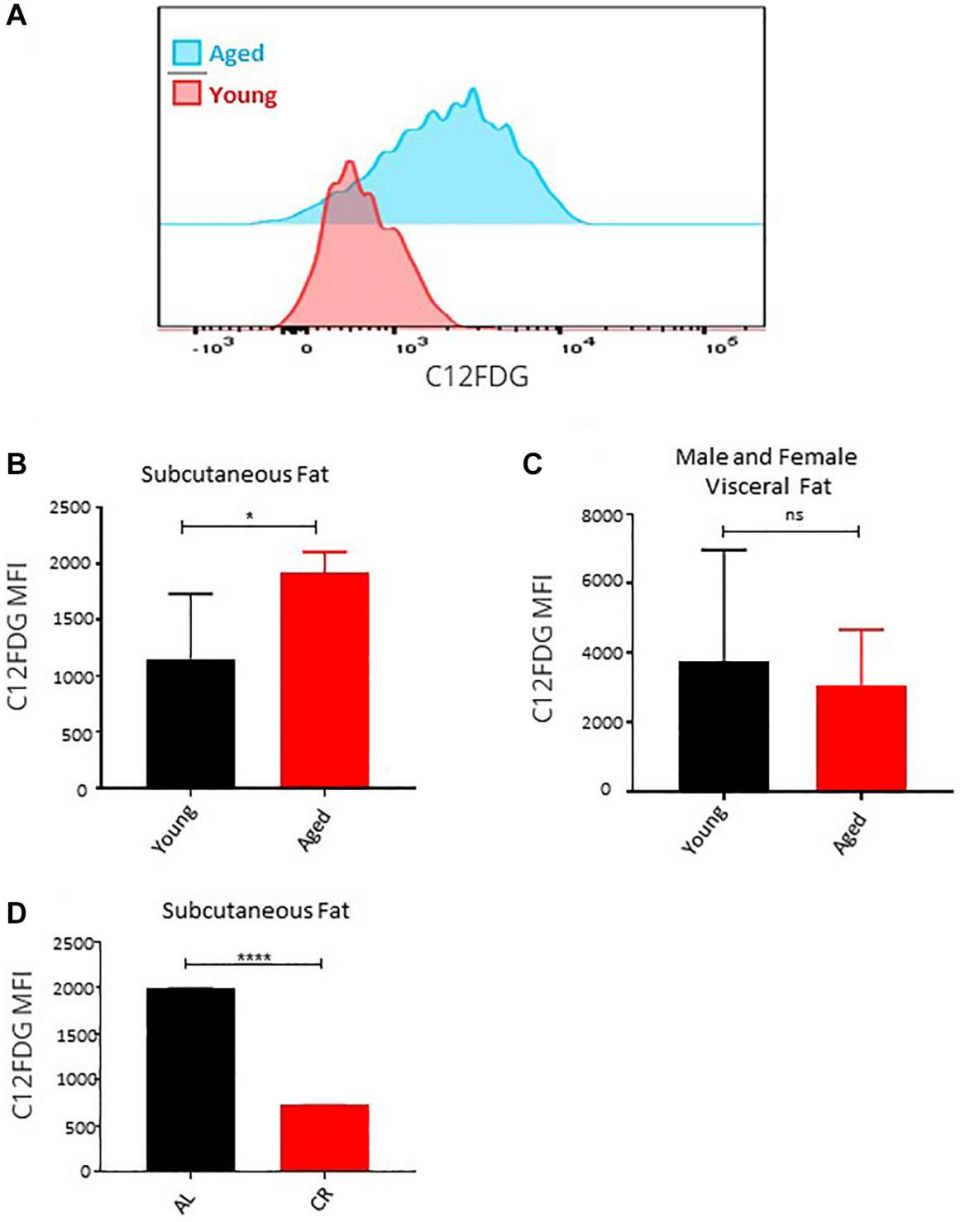
**Effect of age on senescence in ASCs.** (**A**) Representative histogram of senescence marker C12FDG fluorescence among ASCs from young and aged mice. (**B**, **C**) The mean fluorescence intensity distribution of C12FDG in subcutaneous (**B**) and visceral (**C**) ASCs from young and aged mice. Graphs are representative of eight mice per group and ASCs from two mice were pooled together to achieve 5000 events count. (**D**) The mean fluorescence intensity distribution of C12FDG in subcutaneous ASCs from mice fed on caloric restricted and ad-libitum diet. (*n*= 3–5 mice per group) *P* value < 0.05 = ^*^, <0.00005 = ^****^, ns = non-significant.

Senescent cells accumulate in adipose tissue during aging because of replicative, inflammation, and metabolic stresses [40]. Senescent ASCs have a lower adipogenesis differentiation capacity and induce senescence in bystander ASCs through SASP secretion [41]. Using fluorescent substrate C12FDG-based assay that determine the senescence-associated β-galactosidase activity in cells, we determined the presence of senescence cells in the subcutaneous and visceral adipose tissue of male and female young and aged mice *ex vivo*. Flow cytometric evaluation of the C12FDG fluorescence revealed a significantly higher β-galactosidase activity in the ASCs isolated from subcutaneous adipose tissue of the aged mice (Figure 5A, 5B). While in line with our observation showing no effect of age on visceral adipose ASCs, the senescence marker assay showed no significant change (Figure 5C). Long-term caloric restriction prevented the accumulation of senescent cells in the subcutaneous adipose tissue (Figure 5D). We conclude that senescent cells accumulate with age in subcutaneous fat and reduced caloric intake for a longer period helps reduce the accumulation of senescent cells.

### Aging results in loss of autophagy activity in subcutaneous fat resident ASCs that can be restored by long-term caloric restriction

**Figure 6:**
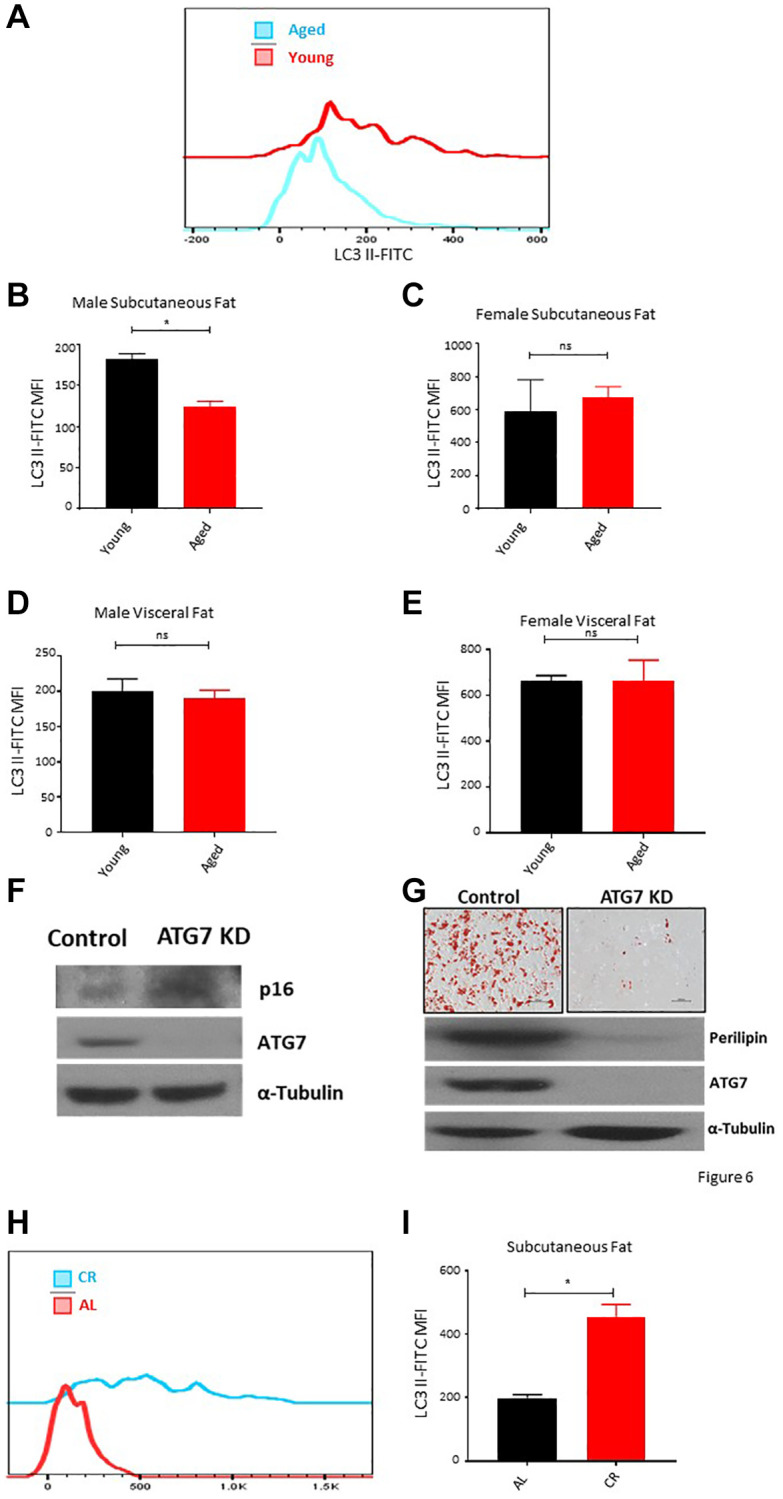
**Aging is associated with autophagy defects in ASCs.** (**A**) Representative histogram of autophagy marker LC3 II fluorescence among ASCs from young and aged mice. (**B**, **C**) The mean fluorescence intensity distribution of LC3 II in subcutaneous ASCs from young and aged male (**B**) and female (**C**) mice. (**D**, **E**) The mean fluorescence intensity distribution of LC3 II in visceral ASCs from young and aged male (**D**) and female (**E**) mice. (**F**) Western blot of ASCs lysate from control and ATG7 KO mice. Blots were developed for p16, ATG7, and α-Tubulin. (**G**) ASCs isolated from control and ATG7 KO mice were differentiated and stained with Oil-Red-O. Differentiated ASCs lysate was blotted for perilipin, ATG7, and α-Tubulin expression. (**H**, **I**) The mean fluorescence intensity distribution of LC3 II in subcutaneous ASCs from mice fed on caloric restricted and ad-libitum diet. (*n* = 3–5 mice per group) *P* value < 0.05 = ^*^, ns = non-significant.

Autophagy is a constitutive pathway that controls the quality of the proteome by recycling damaged protein in the lysosome [42, 43]. It is a critical process to maintain cellular homeostasis under stress conditions and has been widely studied as an anti-aging mechanism [44]. We utilized a flow cytometry-based assay to access the autophagy activity in ASCs *ex vivo*. Results showed a decrease in the autophagy activity depicted by lower LC3-II levels in subcutaneous ASCs of aged male mice (Figure 6A, 6B), while no noticeable autophagy changes were observed in ASCs isolated from the aged female mice subcutaneous adipose tissue (Figure 6C). Furthermore, ASCs isolated from the visceral adipose tissue of young and aged mice also showed no significant autophagy activity changes in both sexes (Figure 6D, 6E). To establish a correlation between autophagy activity, senescence, and loss of differentiation capacity, we isolated subcutaneous ASCs from ATG7 KD mice and analyzed the senescence marker p16 expression (Figure 6F) and their differentiation capacity (Figure 6G). Western blot analyses of ATG7 KD ASCs showed an increase in p16 expression compared to the control (Figure 6F). ATG7KD ASCs showed defective differentiation capacity reflected by lower Oil-Red-O positive cells and lower perilipin protein expression of differentiated cells (Figure 6G). These results confirmed that aging is associated with a decrease in autophagy activity in subcutaneous ASCs and these cells present senescence features and poor differentiation potential. Next, we analyzed whether long-term caloric restriction can prevent the loss of autophagy activity in subcutaneous ASCs. Flow cytometry results demonstrated that mice undergoing long-term caloric restriction have a significantly higher autophagy activity compared to aged match ad-libitum controls (Figure 6H, 6I).

## DISCUSSION

The process of aging is marked by gradual physiological deterioration and stands as the leading contributor to global human pathologies and mortality. Among the tissues susceptible to aging, adipose tissue emerges as particularly vulnerable, undergoing disruptions in various biological and physiological processes that consequently impact overall health. Within the context of aging, malfunctioning adipose tissue gives rise to persistent low-grade inflammation, insulin resistance, and infiltration of lipids in visceral organs. This investigation delves into the alterations manifesting in the distribution of subcutaneous and visceral adipose tissue compartments as individuals age, focusing specifically on adipose-derived stem cells residing within these compartments. Our study involves a comparative analysis of oxidative stress levels, mitochondrial activity, expression of senescence markers, and autophagy in adipose-derived stem cells residing in the subcutaneous and visceral adipose tissue of both young and aged mice.

During the aging process, significant alterations occur in the mass and distribution of adipose tissue. The accumulation of total fat mass is a common occurrence among both healthy and unhealthy older individuals, often emerging as early as middle age [32]. Another transformation linked to aging is the redistribution of body fat, characterized by a prominent rise in visceral fat, coupled with a decrease in subcutaneous fat in the lower body [45]. Subcutaneous and visceral adipose depots differ markedly in terms of their metabolic effects. Generally, subcutaneous adipose tissue (SAT) is deemed metabolically advantageous, whereas visceral adipose tissue (VAT) is considered detrimental. The age-related shift in adipose tissue distribution toward visceral depots profoundly impacts overall healthy aging. Consequently, the rearrangement of fat distribution during aging is associated with an elevated susceptibility to metabolic irregularities, notably insulin resistance, which correlates with an increased risk of cardiovascular disease and diabetes [32]. We observed a very similar trend in our analyses of young and aged, male and female mice. We observed a significant decrease in subcutaneous to total body mass, particularly in male mice, and visceral adipose depot mass increase in both sexes with age. Similarly, we observed a significant increase in the visceral to subcutaneous fat ratio in male mice. Intriguingly, long term caloric restriction results in a significant reversal of the fat distribution and results in a significant decrease in the visceral to subcutaneous fat ratio [46].

While adipose tissue dysfunction is extensively documented in obesity-related metabolic disorders, its contribution to age-related metabolic dysfunction remains less understood. Elevated fibrosis levels are a defining feature of metabolically compromised adipose tissue, believed to constrain adipocyte lipid storage capacity and promote lipid redistribution to ectopic sites. In our study, we observed a marked increase in adipose tissue fibrosis with age in both male and female mice, consistent with previous findings demonstrating age-related fibrosis escalation [47]. Age-related alterations in adipocyte size are also implicated, with ectopic lipid accumulation often associated with compromised pre-adipocyte differentiation to mature adipocytes [47]. Our investigation revealed no significant changes in adipocyte size among aged male or female mice. Corrales et al. similarly noted stable adipocyte sizes between young (3 months) and aged (12 months) mice, whereas Donato et al. reported a notable decrease in adipocyte size in very aged mice (29 months old) [46, 47]. These findings delineate a dynamic shift in adipocyte morphology across age groups, characterized by size increases in middle age followed by continuous reductions in older age [20]. Interestingly, long-term caloric restriction elicited a significant reduction in adipocyte size, suggesting its potential role in modulating age-related adipose tissue alterations [46]. These insights underscore the complex interplay between age, adipose tissue dynamics, and metabolic regulation, highlighting the potential impact of dietary interventions on adipocyte morphology and function.

Aging induces not only anatomical changes but also significantly impacts the metabolic function of adipose tissue. Extensive studies examining the effects of aging and caloric restriction on metabolic alterations in adipose tissue have been conducted [46–48]. These studies revealed no significant changes in non-fasting glucose levels between young and aged mice. However, aged mice exhibited higher fasting glucose and insulin levels compared to their younger counterparts [46, 47]. Moreover, aged mice demonstrated significant glucose intolerance upon challenge, indicating metabolic dysregulation associated with aging [46, 47]. Notably, a substantial increase in fasting insulin and HOMA-IR in 12-month-old mice suggests the onset of insulin resistance during middle age, implying that adipose tissue changes contributing to metabolic dysregulation in old age may commence between young and middle age and worsen with aging [46]. Additionally, aged mice exhibited higher mitochondrial respiratory oxygen flux and increased abundance of nitrotyrosine, indicative of tissue oxidative stress [47]. Furthermore, aging altered the adipose tissue microenvironment, leading to changes in the intrinsic chemical properties of NAD(P)H [48].

Adipose stem cells (ASCs) are integral to the regeneration of adipose tissue. They undertake self-replication and differentiation, which in turn supports the maintenance of the stem cell reserve in the tissue. ASCs are distinguished by their impressive capacity to multiply and transform into fat cells, thereby facilitating the repair or replacement of damaged or diminished fat tissue. However, this property of stemness in ASCs is detrimentally affected by both aging and obesity [33]. As people grow older, there is a noticeable decrease in ASCs’ ability to rejuvenate themselves and turn into fat cells, which is characterized by reduced proliferation potential, altered gene expression, and hindered differentiation capabilities [17]. These changes related to aging lead to a general decline in the performance of adipose tissue, potentially leading to aging-associated metabolic disorders [21]. The transformation of ASCs into fat cells results in increased energy requirements and metabolic rates, escalating mitochondrial activity and the generation of reactive oxygen species (ROS). Higher levels of ROS can push stem cells towards differentiation, culminating in senescence and cell death [49]. In our study, we executed an in-depth *ex vivo* study of ROS production and mitochondrial activity in adipose tissue segments of young and older mice. By using mitochondrial membrane potential as a stemness indicator, our findings suggest that aging is linked with the loss of stemness in ASCs in subcutaneous adipose compartments, leading to higher ROS production and oxidative stress. This finding likely accounts for the inability of subcutaneous adipose tissue to grow with age. Interestingly, ASCs from visceral fat do not show significant differences in ROS levels and mitochondrial activity, suggesting that ASCs in visceral depots maintain their proliferation and differentiation abilities, aiding tissue expansion even as they age. Further studies based on more sophisticated molecular tagging of ASCs *in vivo* are required to confirm the effect of aging on oxidative stress and loss of quiescence. In addition, more careful interpretation of data derived from *in vivo* ASCs vs. cell cultured ASCs is warranted as culturing cells on plastic change many of the metabolic dynamics.

Cellular senescence, a condition marked by cell division arrest, is linked to a decrease in the regenerative capacity and functionality of various tissues, contributing to the overall aging process [32]. Adipose tissue, or fat, is a significant location for the buildup of senescent cells with age, triggered by a mix of replication, cytokine-induced, and metabolic stresses. While cellular senescence is thought to be a protective mechanism against cancer, its presence in adipose tissue can lead to numerous issues, such as impaired fat cell formation, inflammation, abnormal adipocytokine production, and insulin resistance. Senescent cells release a mixture of cytokines, chemokines, proteases, and growth factors known as the SASP, which is seen as a signal of aging [32]. The reduced stemness and differentiation in aged ASCs could also be due to the buildup of these senescent cells. Research has shown that senescent fat cell progenitors in humans can inhibit the fat cell formation of nearby non-senescent progenitors through a paracrine pathway [50]. When these progenitors are cultured with senescent cells, only 20% accumulate lipids, compared to over 50% when cultured with non-senescent cells [41]. This effect could be linked to activin A, interleukin-6 (IL-6), TNF-α, interferon-γ (IFN-γ), and/or the SASP components of senescent fat cell progenitors or other types of senescent cells. Consistent with existing research, we too noticed a buildup of senescent cells in the subcutaneous fat tissue compartments of older subjects. Interestingly, we did not see any variation in the distribution of senescence markers in the adipose stem cells (ASCs) found in visceral fat tissues, highlighting the unique behavior and traits of these visceral fat ASCs. The distinct nature of visceral fat ASCs observed in our study suggests a potential difference in the origin of ASCs located in subcutaneous and visceral tissues. This could explain the variations in the biological characteristics of mature fat cells derived from these progenitors.

Several processes have been identified that trigger inflammation as we age. One such process is the misregulation of autophagy activity in aging adipose tissue, which leads to increased endoplasmic reticulum (ER) stress and inflammation. The ER stress response in aging adipose tissue contributes to age-related inflammation [32]. Our *ex vivo* analyses demonstrated that aged subcutaneous ASCs have lower autophagy activity compared to their younger counterparts while similar to the other metabolic and biochemical patterns observed in our analyses for the visceral ASCs, autophagy activity was not affected by age. To demonstrate that the decrease in autophagy is responsible for the senescence and loss of stemness in ASCs [25, 26], we confirmed higher p16 expression and lower differentiation in ASCs isolated from ATG7 KO mice.

Caloric restriction (CR), a method involving a chronic reduction in total calorie intake without inducing malnutrition, is widely employed to prevent excessive fat accumulation and prolong the health span across various organisms. CR effectively mitigates age-related adipose tissue buildup, which can detrimentally affect neighboring or distant organs. Moreover, CR, in conjunction with nutrient deprivation, triggers appropriate levels of autophagy, leading to the removal of dysfunctional organelles, proteins, and aggregates from the cytoplasm [25]. This process involves the regulation of key genes such as AMP-activated protein kinase (AMPK). In our investigation, we explored the impact of long-term caloric restriction on adipocytes and ASCs. Our findings indicate that CR enables mice to counteract the detrimental effects of aging on ASC biology. CR promoted heightened autophagic activity and reduced the accumulation of senescent ASCs, indicative of enhanced stemness features. Prior studies have underscored the advantageous effects of caloric restriction in delaying age-related insulin resistance, reducing tissue fibrosis, and mitigating inflammation [46]. While our study focuses on the effects of long-term caloric restriction on adipose tissue biology, short-term caloric restriction (3–6 weeks) has demonstrated beneficial effects in reducing body mass, adipose tissue mass, glucose levels, and energy expenditure [27]. Notably, short-term caloric restriction over four months significantly decreased adipocyte size and the expression of inflammatory genes in male rhesus macaques [51]. Further investigations are warranted to assess the impact of short-term caloric restriction on ASC biology across different age groups.

In conclusion, our results demonstrate that aging results in the accumulation of oxidative stress and senescent ASCs in subcutaneous adipose tissue leading to the loss of stemness. The loss of autophagy activity is one of the contributing factors to age-associated deterioration in ASCs function. Long-term caloric restriction enables subcutaneous ASCs to resist age-associated changes. In the future, studies involving caloric restriction for a shorter duration, intermittent fasting, and caloric restriction mimetics drugs with focused read-outs on ASCs biology will enable the translation of these results to achieve the goal of healthy aging.

## Supplementary Materials

Supplementary Figure 1
