# Supplementary material for: Caloric restriction mitigates age-associated senescence characteristics in subcutaneous adipose tissue-derived stem cells
Source: Aging (Albany NY). 2024 May 9;16(9):7535–52. doi: 10.18632/aging.205812 (PMC11131987; doi:10.18632/aging.205812)
Supplement: Supplementary Figure 1 [file aging-16-205812-s001.pdf]

SUPPLEMENTARY FIGURE

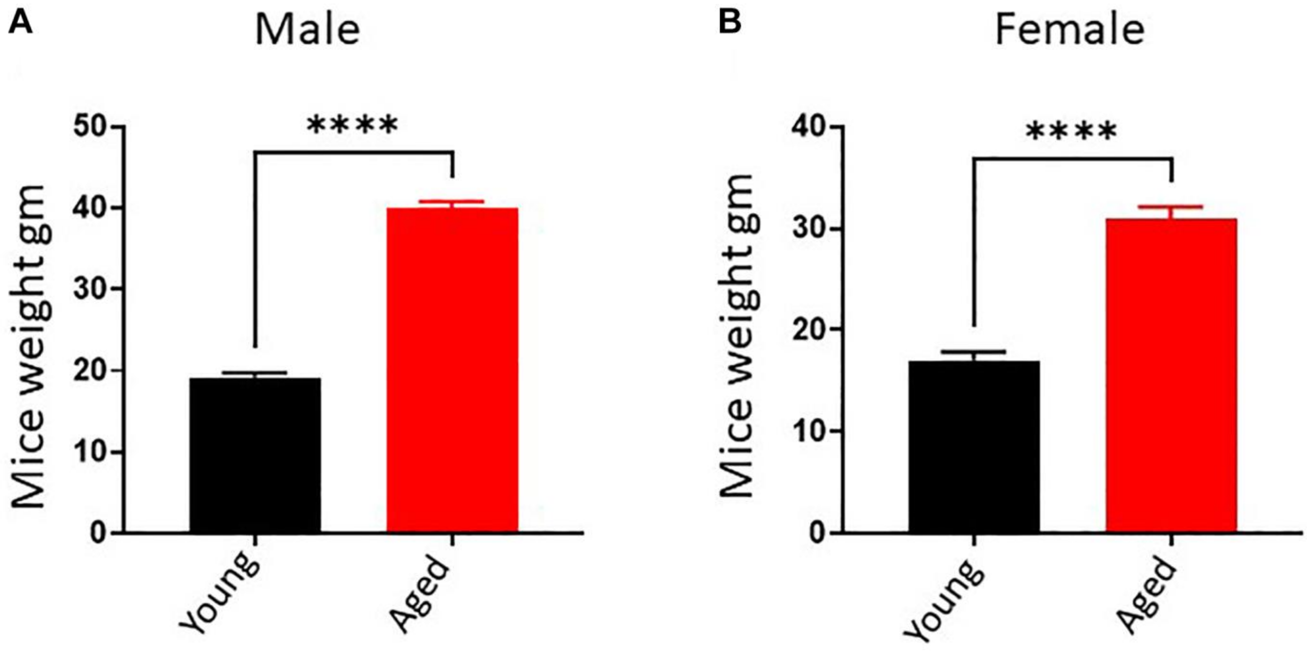

Supplementary Figure 1. Weight of young and aged male (**A**) and female (**B**) mice. *P*-value < 0.00005 = ****.
